# PCSK9 Functions in Atherosclerosis Are Not Limited to Plasmatic LDL-Cholesterol Regulation

**DOI:** 10.3389/fcvm.2021.639727

**Published:** 2021-03-23

**Authors:** Aureli Luquero, Lina Badimon, Maria Borrell-Pages

**Affiliations:** ^1^Cardiovascular Program ICCC, IR-Hospital de la Santa Creu i Sant Pau, IIB-Sant Pau, Barcelona, Spain; ^2^Centro de Investigación en Red- Área Cardiovascular, Instituto de Salud Carlos III, Madrid, Spain; ^3^Cardiovascular Research Chair, Universitat Autònoma de Barcelona, Barcelona, Spain

**Keywords:** atherosclerosis, PCSK9 (proprotein convertase subtilisin kexin type 9), lipoprotein receptors, inflammation, lipid loading, LDL—cholesterol

## Abstract

The relevance of PCSK9 in atherosclerosis progression is demonstrated by the benefits observed in patients that have followed PCSK9-targeted therapies. The impact of these therapies is attributed to the plasma lipid-lowering effect induced when LDLR hepatic expression levels are recovered after the suppression of soluble PCSK9. Different studies show that PCSK9 is involved in other mechanisms that take place at different stages during atherosclerosis development. Indeed, PCSK9 regulates the expression of key receptors expressed in macrophages that contribute to lipid-loading, foam cell formation and atherosclerotic plaque formation. PCSK9 is also a regulator of vascular inflammation and its expression correlates with pro-inflammatory cytokines release, inflammatory cell recruitment and plaque destabilization. Furthermore, anti-PCSK9 approaches have demonstrated that by inhibiting PCSK9 activity, the progression of atherosclerotic disease is diminished. PCSK9 also modulates thrombosis by modifying platelets steady-state, leukocyte recruitment and clot formation. In this review we evaluate recent findings on PCSK9 functions in cardiovascular diseases beyond LDL-cholesterol plasma levels regulation.

## Introduction

Proprotein Convertase Subtilisin/Kexin Type 9 (PCSK9) is a soluble protein synthesized as a zymogen that undergoes autocatalytic cleavage in the endoplasmic reticulum (1). In 2007, PCSK9 was found to be a ligand for Low Density Lipoprotein Receptor (LDLR) a key cell membrane receptor in cholesterol homeostasis regulation (2). LDLRs bind and internalize low density lipoproteins (LDL) from the bloodstream, clearing the blood from highly-enriched cholesterol lipoproteins. The LDL-LDLR complex is guided to the lysosome where LDLs are digested and LDLRs are recycled to the cell surface to keep clearing LDL particles from the circulation. PCSK9 inhibits LDLR recircularization by promoting its degradation in the lysosomes along with LDLs (3). This effect highly reduces the presence of LDLR at the hepatocyte's cell surface and consequently, there is an increase in LDL particles in the bloodstream.

Although PCSK9 is known since 2003 (4) and was almost immediately associated with hypercholesterolemia (5), the knowledge of its potential role on LDL metabolism regulation and its associated diseases is increasing over the years (6, 7). It was first described that mutations on PCSK9 that lead to gain-of-function variants of the protein were responsible for different cases of human familial hypercholesterolemia [FH; (8, 9)]. FH is an inherited disease where patients have LDL plasma levels above 190 mg/dL, contributing to an elevated risk of atherosclerotic plaque formation and coronary adverse events. Contrarily, PCSK9 loss-of-function mutations are associated with very low levels of LDL in blood reducing the cardiovascular associated risk (10, 11). These data encouraged studies to test if PCSK9 was a good target for clinical trials to treat hypercholesterolemia. Hypercholesterolemia is commonly treated with statins, which are drugs that inhibit HMG-CoA reductase, a key enzyme for cholesterol biosynthesis that reduces cholesterol production and lowers LDL concentration in plasma. However, some patients present statin intolerance which hampers the treatment (12). Both PCSK9 and LDLR gene expression are regulated by SREBP2 [Sterol Regulatory Element-Binding Protein 2; (13–15)]. When intracellular levels of cholesterol are low (as after statin treatments), there is activation of SREBP2 that promotes PCSK9 and LDLR transcription. Therefore, both LDLR and LDLR's inhibitor protein levels increase resulting in an intrinsic loop that limits statin therapy efficacy (16–18). SREBP2 transcriptional activity is regulated upstream by AMPK (AMP-activated protein Kinase). Activation of AMPK leads to SREBP2 phosphorylation and its inability to promote transcription of target genes (19, 20).

Anti-PCSK9 drugs started to be developed as a secondary approach to reduce LDL cholesterol levels in hypercholesterolemic patients. To date, only two monoclonal antibodies targeting PCSK9 are available for treating hypercholesterolemia: evolocumab and alirocumab. They were tested in the OSLER trial (21) and in the ODYSSEY LONG TERM trial (22), respectively (Table 1). Both studies showed a ~60% decrease of LDL particles in blood and a decrease in cardiovascular events including myocardial infarction, unstable angina or stroke (2.18% in placebo and 0.95% in evolocumab-treated patients in the OSLER trial and 5.1% in placebo and 4.6% in alirocumab-treated patients in the ODYSSEY LONG TERM study). However, the cardiovascular events reported during these studies were too low to demonstrate clinical relevance in this area. The FOURIER trial enrolled patients with previous atherosclerotic cardiovascular disease that were on statin therapy (23). Results showed a 59% reduction of LDL content in bloodstream and decreased cardiovascular events including cardiovascular mortality, myocardial infarction, stroke, hospitalization for unstable angina or coronary revascularization by more than 15% in evolocumab treated patients after 26 months follow-up. The ODYSSEY OUTCOMES study in patients with recent acute coronary syndrome at maximum tolerated dose of statins showed that alirocumab administration was associated with a reduced risk of recurrent ischemic cardiovascular events and also with a reduced mortality (24). Finally, the SPIRE 1 and SPIRE 2 trials were randomized trials that compared the efficacy of bococizumab, another anti-PCSK9 antibody, with placebo in patients that suffered previous cardiovascular events. These studies did not show benefits from bococizumab treatment despite showing significant improvements for patients with a high cardiovascular risk. There was reduced clinical efficacy because half of patients receiving bococizumab therapy developed antidrug antibodies probably because bococizumab is a murine humanized antibody containing approximately a 3% of murine sequence. Contrarily, alirocumab and evolocumab are antibodies with full human sequence. The negative results concluded in a premature stop of the trial by the sponsor (25).

**Table 1:** Study characteristics and outcomes of clinical trials with monoclonal antibodies against PCSK9.

	**Dosing**	**Patients/Treatment ratio**	**Inclusion criteria**	**Results**	**Study limitations**
**OSLER TRIAL**					
• OSLER-1: open-label, randomized and controlled study of patients from Phase II Evolocumab trials • OSLER-2: open-label, randomized and controlled study of patients from Phase III Evolocumab trials	420 mg/month or 140 mg/2 weeks	2:1 2,976 patients on Evolocumab : 1,489 patients on previous treatment (± statins)	• No adverse events in previous evolocumab studies. • Not having unstable medical condition. • Not expected to need adjustments of background lipid-regulating therapy.	• 61% reduction in LDL levels • 56% reduction in adverse CVE	• Open-label design • Low number of adverse CVE • Only patients who did not suffer CVE during previous Evolocumab therapy were accepted • High variability in patients' cardiovascular risk and use of statins
**ODYSSEY LONG TERM**					
A Phase III, randomized, double-blind, placebo-controlled, parallel-group and multinational study	150 mg/2 weeks	2: 1 1,553 patients on Alirocumab : 788 patients on placebo	• Heterozygous FH, coronary heart disease or equivalent risk • LDL-cholesterol levels above 70 mg/dL at screening • Patients under high-dose statin therapy or maximum-tolerated dose	• 62% reduction in LDL levels • 48% reduction adverse CVE	• Short follow-up period for a chronic disease evaluation (20 months). • Low number of CVE, limiting the robustness of the data.
**FOURIER TRIAL**					
Randomized, double-blinded, placebo-controlled, multicenter trial	140 mg/2 weeks or 420 mg/month	1: 1 13,784 patients on Evolocumab : 13,780 patients on placebo	• ≥40 and ≤ 85 years-old • Clinical evidence of atherosclerotic cardiovascular disease • LDL cholesterol ≥ 70 mg/dL, non-HDL cholesterol ≥ 100 mg/dL while on lipid lowering therapy	• 59% reduction in LDL cholesterol after 42 weeks • 15% reduction in CVE after 26 months	Median of 2,2 years
**ODYSSEY OUTCOMES**					
Randomized, double-blinded, placebo-controlled, multicenter trial	75 mg/2 weeks	1: 1 9,462 Patients on Alirocumab : 9,462 patients on placebo	• ≥40 years old • Hospitalization 1 ≤ and ≥ 12 months with acute coronary syndrome • LDL cholesterol ≥ 70 mg/dL, non-HDL cholesterol ≥ 100 mg/dL and apoB ≥ 80 mg/dL	• 54,7% reduction in LDL cholesterol after 48 months • 15% reduction of CVE and 15% reduction of death	Median of 2,8 years
**SPIRE-1 and SPIRE-2**					
• Spire-1 patients were eligible with at least 70 mg/dL of LDL cholesterol at screening • Spire-2 patients were eligible with at least 100 mg/dL of LDL cholesterol at screening	150 mg/2 weeks	1: 1 13,720 Patients on Bococizumab : 13,718 patients on placebo	• Men ≥ 50/Women ≥ 60, in case of FH Men ≥35/Women ≥ 45 • Previous CVE or a history of diabetes, chronic kidney disease or peripheral vascular disease with cardiovascular risk or familial hypercholesterolemia • Additional risk factors • On statin-therapy unless completely intolerance to statins is presented.	• 59% reduction in LDL cholesterol after 14 weeks • 12% reduction of CVE incidence	Median of 10 months (the study was not finished)

*LDL, low density lipoprotein; HDL, high density lipoprotein; FH, familial hypercholesterolaemia; apoB, apolipoprotein B; CVE, cardiovascular event*.

Besides monoclonal antibodies, other approaches that target PCSK9 have been developed. Inclisiran (a small interference RNA) and statin administration in patients with atherosclerotic cardiovascular disease or heterozygous hypercholesterolemia patients reduced PCSK9 levels in blood and LDL-cholesterol levels in ORION-9 and ORION-10/ORION-11 phase 3 clinical trials (26, 27).

Regulation of cholesterol-rich LDL blood levels is not the only role that PCSK9 drives in atherosclerosis pathogenesis. There is a strong background suggesting alternative roles for PCSK9 in the development of atherosclerosis (28, 29). Indeed, a prospective cohort of 4.232 sixty-year-old men and women living in Stockholm County showed that independently of LDL plasma levels, PCSK9 levels correlate with elevated probability of future cardiovascular events (30). In another study that included 643 participants, high plasma PCSK9 levels correlated with enhanced atherosclerosis progression independently of LDL, as measured by carotid plaque formation and total plaque area (31). However, prospective studies have failed to demonstrate a relationship between PCSK9 expression levels and future risk of cardiovascular events despite revealing correlations between PCSK9 plasma levels and atherosclerotic markers including LDL-cholesterol, blood triglycerides or insulin (32, 33).

This review will discuss the role of PCSK9 in modulating the activity of different cell lineages involved in atherosclerosis progression including macrophages, vascular smooth muscle cells (VSMC), endothelial cells (EC), lymphocytes and platelets and its associated cardiovascular risk. We will comment on PCSK9 effector roles showing that they extend far beyond the regulation of LDL particles and reveal new insights by which PCSK9 inhibitors may lower the incidence of atherosclerosis progression.

## PCSK9 Modulates Macrophage's Lipid-Uptake Receptors

**Figure 1:**
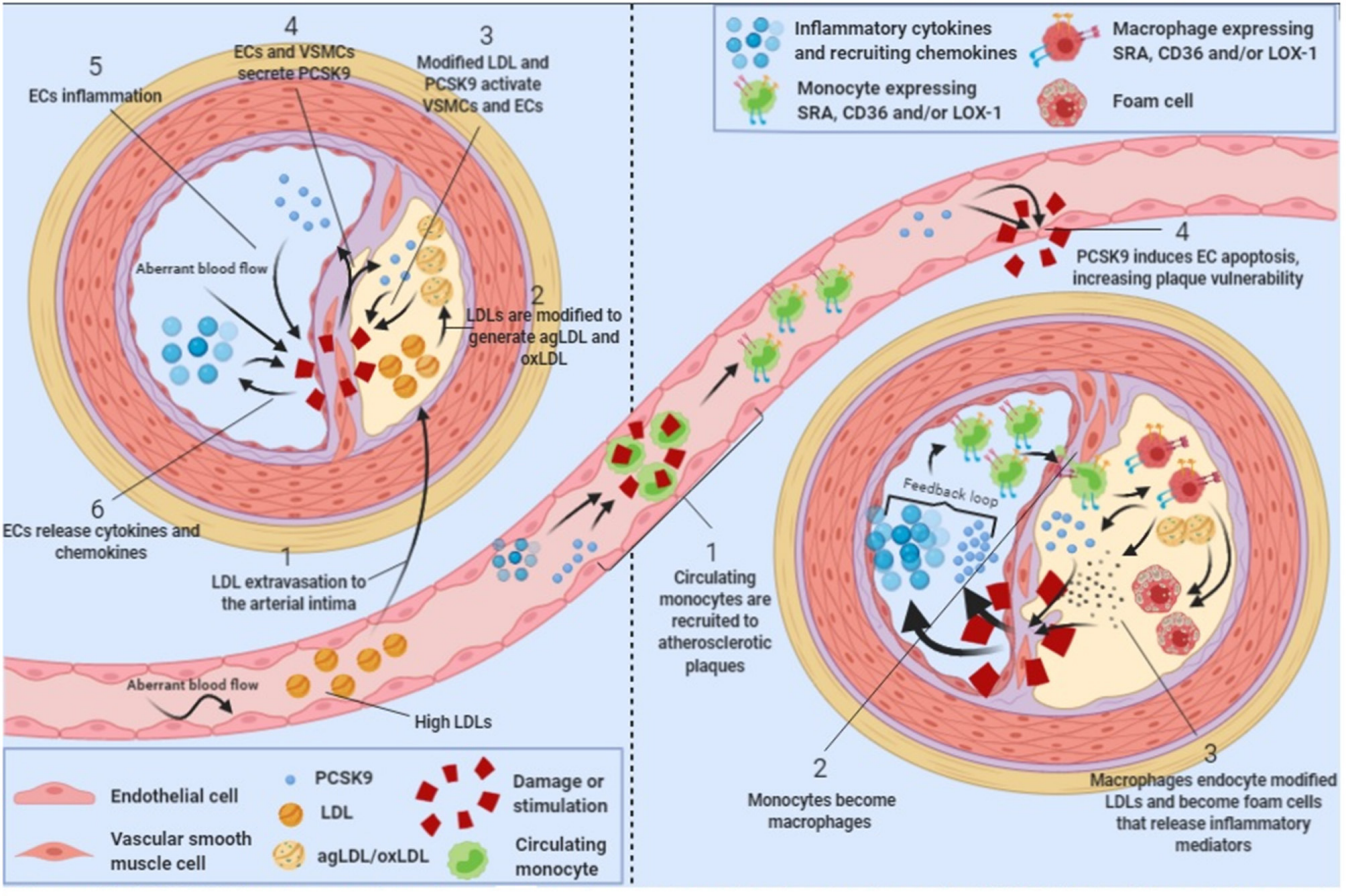
PCSK9 in atherosclerosis progression. Schematic showing the role of PCSK9 in different stages of atherosclerosis progression.

Atherosclerosis is commonly described as a chronic inflammatory disease that starts with an excess of cholesterol accumulation in the vascular wall triggering inflammation (34). Macrophages are inflammatory cells that play a key role in lipid uptake and atherosclerosis progression. In 2012, it was shown that LDLR expressed in the surface of human macrophages were downregulated by PCSK9 produced by VSMC, reducing the ability of macrophages to internalize native LDL molecules and avoiding the formation of foam cells, indicating that PCSK9-stimulated macrophages reduce foam cells formation and hence, reduce atherosclerosis progression (35). However, native LDL molecules are not the major source of cholesterol accumulation in macrophages. Upon vascular extravasation, LDL molecules undergo several modifications including aggregation and oxidation. LDL aggregation and oxidation occur after extracellular matrix components such as glycosaminoglycans (36) or chondroitin sulfate proteoglycans (37) retain native LDL particles and facilitate their modification by several secreted enzymes including secretory phospholipase A2, sphingomyelinase, lipoxygenase or myeloperoxidase (38–40). Modified LDL particles generate aggregated (agLDL) and oxidized (oxLDL) LDLs, which are the major source of cholesterol ester accumulation in macrophages and VSMCs [Figure 1; (41–44)]. Macrophages do not internalize agLDL or oxLDL through LDLR but through a different group of receptors called scavenger receptors (45) and LDLR related proteins [LRPs; (46–48)].

Scavenger receptors including scavenger receptor A (SRA), cluster of differentiation 36 (CD36) and lectin-like oxidized low-density lipoprotein receptor 1 (LOX-1) promote the endocytosis of oxLDL particles in monocytes and macrophages and their expression is highly increased under different inflammatory stimulus including lipopolysaccharide (LPS) or tumor necrosis factor-α [TNFα; Figure 1; (49, 50)]. Main features of scavenger receptors are summarized in Table 2. LPS are major components of the outer membrane of Gram-negative bacteria that are recognized by Toll-Like Receptor 4 (TLR4) expressed in macrophage's cell surface. Binding of LPS to TLR4 triggers an intracellular response that activates both MAPK and NFκB pathways triggering inflammation. PCSK9 expression levels are increased in mouse macrophages after LPS stimulation as a result of the activation of the NLRP3 (NOD-Like Receptor Protein 3) inflammasome. Indeed, NLRP3 and its downstream signals IL-1β, IL-18, and caspase 1 all participate in PCSK9 secretion as confirmed by specific gene deletion experiments (51).

**Table 2:** Scavenger Receptors and LDLR main features.

	**LOX-1**	**SRA**	**CD36**	**LDLR**
Cell expression	• VSMCs, endothelial cells, macrophages, platelets, fibroblasts	• Macrophages, VSMCs, endothelial cells	• Macrophages, monocytes, platelets, endothelial cells, erythrocytes	• Particularly elevated in hepatocytes • Ubiquitous expression
Upon PCSK9 stimulation	↑ expression	↑ expression	↑ expression	↓ expression
Deficiency	• Reduced oxLDL uptake in macrophages • Atheroprotective and anti-inflammatory	• Reduced oxLDL uptake in macrophages • Reduced inflammatory response • Macrophage apoptosis	• Reduced oxLDL uptake in macrophages • Atheroprotective	• Responsible for FH
Functions	• Pro-atherogenic • Pro-inflammatory • Pro-thrombotic • Induces PCSK9 expression in VSMCs • Endocytosis of oxLDL • Endothelial dysfunction • Foam cell formation • Macrophages, VSMC, endothelial cell apoptosis	• Pro-atherogenic • Pro-inflammatory • Endocytosis of oxLDL • In antigen presenting cells, mediates pathogen phagocytosis	• Pro-atherogenic • Pro-inflammatory • Pro-thrombotic • Endocytosis of oxLDL • Inhibits macrophage migration • Promotes platelet activation/aggregation	• Atheroprotective • Endocytosis of nLDL
Other regulations	Upregulated in VSMCs, macrophages and monocytes during oxidative stress and inflammation	Upregulated in VSMCs and endothelial cells during oxidative stress Upregulated in macrophages and monocytes during inflammation	Upregulated in macrophages by fat-rich diets, inflammation and oxidative stress	

*nLDL, native low density lipoproteins; oxLDL, oxidized low density lipoproteins; VSMCs, vascular smooth muscle cells; FH, familial hypercholesterolemia*.

PCSK9 expression in macrophages after TNFα stimulation relies on the generation of reactive oxygen species (ROS). ROS inhibitors diphenyleneiodonium (DPI) and apocynin reduce PCSK9 expression while ROS inducers pyocyanin and antimycin A increase PCSK9 release showing that PCSK9 is expressed during macrophage proinflammatory procedures (50). ROS production is dependent on NADPH oxidase. Upon TNFα-stimulation lack of different NADPH oxidase complex subunits reduces the amount of scavenger receptors in the surface of macrophages (50). Recombinant PCSK9 administration increases SRA, CD36, and LOX-1 both at gene and protein levels in cultured mouse macrophages. Concomitantly, oxLDL uptake is increased (Figure 1). This increased lipid uptake is abolished in macrophages that lack SRA, CD36, or LOX-1 suggesting that all three receptors are involved in oxLDL uptake and consequently, in the generation of foam cells in atherosclerosis (50). Table 2 summarizes the involvement and regulation of scavenger receptors and LDLR in different processes associated with atherosclerosis.

Other cell surface receptors expressed in macrophages modulated by PCSK9 are LRP1, LRP5, and LRP8. These receptors belong to the LRP subfamily of the LDLR superfamily of receptors and conserve the characteristic EGF domain that allows PCSK9 binding (52). LRP1 surface levels, together with LDLR, are downregulated by human PCSK9 in atherosclerotic mouse macrophages inducing increased gene expression of the proinflammatory markers TNFα and IL-1β and decreased gene expression of the anti-inflammatory markers IL-10 and arginase-1 indicating enhanced macrophage polarization toward a pro-inflammatory phenotype (53). LRP8 (aka apoER2) a receptor known for recognizing ApoE protein is also downregulated upon recombinant PCSK9 binding in different cell lines including HEK293, 3T3 fibroblasts, CHO, NeuroA2 and HuH7 (54). We have recently described that LRP5 is required for lipid internalization in human macrophages as in the absence of PCSK9 and/or LRP5, macrophages show reduced cholesterol ester accumulation (55). Both proteins form a complex at the perinuclear area of human macrophages that immunoprecipitate together. Their interaction is stronger in lipid loaded macrophages (55). In addition, macrophages silenced for LRP5 show reduced release of PCSK9, indicating that LRP5 is involved in soluble PCSK9 release, probably by participating in the intracellular transport of PCSK9 to the plasma membrane (55). Furthermore, we also show that the complex LRP5-PCSK9 up-regulates TLR4/NFκB signaling to favor macrophage inflammation. Interestingly, LRP5 surface levels remain unaltered by secreted PCSK9 (55).

Finally, VLDLR, a receptor that also belongs to the LDLR superfamily and displays a similar structure to that of LDLR is also downregulated by PCSK9 binding. Indeed, treatment of HEK293 cells or 3T3 fibroblasts cells with human recombinant PCSK9 shows a downregulation of VLDLR expression levels (54). Both VLDLR and LRP8 are known to generate anti-inflammatory signaling in macrophages (56).

## PCSK9 Modulates Vascular Inflammation

PCSK9 is mainly produced in liver, kidney and small intestine (4). However, it is also expressed in vascular cells including endothelial cells (ECs) and vascular smooth muscle cells [VSMCs; (35, 57, 58)]. Vascular cells are affected by hemodynamic factors like blood flow that, by inducing wall shear stress, play a critical role in atherosclerosis development and progression (59). Human ECs and VSMCs under low-blood flow have higher PCSK9 protein expression than cells under high blood flow, an effect conserved even after LPS stimulation. Indeed, aortas from *Wt* mice showed significantly higher PCSK9 expression in high shear stress regions, an effect further potentiated by LPS administration (60). Also, in rabbits fed at high-fat diet, low-flow aortic regions had higher PCSK9 expression while regions with high flow such the aortic arch showed lower vascular PCSK9 expression [Figure 1; (61)]. Therefore, there is a negative correlation between PCSK9 vascular expression levels and blood flow.

PCSK9 has been shown to promote vascular inflammation. Binding of PCSK9 to the inflammatory receptor TLR4 was first hypothesized by the structural homology of the C-terminal domain of PCSK9 and the TLR4 ligand resistin in *in silico* simulations (62). TLRs are cell receptors that recognize pathogens and regulate the expression of pro-inflammatory cytokines and also the early immune responses to infection (63). Among TLRs, TLR4 acts as a receptor for LPS and activates NF-κB to promote an inflammatory response (64). PCSK9 expression in ECs and VSMCs is dependent on the TLR4/NFκB signaling pathway as inhibition of different components of the activation cascade show that PCSK9 expression relies on the TLR4-MyD88-NFkB axis and is independent of the TLR4/TRIF signaling, postulating the MyD88 pathway as a possible target for future therapies to prevent excessive PCSK9 production in the vasculature (61). Hence, PCSK9 synthesis is regulated by the TLR4 receptor signaling pathway through MyD88 and NFκB activation, and soluble PCSK9 can act as an inflammatory mediator by TLR4 binding and recognition as demonstrated in *ApoE* knockout mice (65).

Vascular stability depends on cellular apoptosis. PCSK9 modulates the expression of the apoptosis inducer Bax and the apoptosis inhibitor Bcl-2. The balance between these two proteins is key to prevent or trigger apoptosis (66, 67). Lipid loaded endothelial cells show increased Bax protein levels and decreased Bcl-2 levels that lead to caspase 3 and caspase 9 activation inducing cell apoptosis (68). PCSK9 silencing by siRNA, inhibits apoptosis as silenced PCSK9 cannot phosphorylate p38 and JNK (both members of the MAPK signaling pathway) allowing the activation of the apoptosis inhibitor Bcl-2 (69–72). Interestingly, p38 and JNK are also responsible of Bax and Bad phosphorylation that activate programmed cell death (73, 74). Hence, PCSK9 may be promoting MAPK signaling cascade activation and endothelial cell apoptosis [Figure 1], a mechanism that has already been described in cancer cells (75).

## PCSK9 Participates in Plaque Formation

Plaque formation is a complex process that includes lipoprotein retention, inflammatory cells recruitment, VSMC proliferation, matrix synthesis, apoptosis, and necrosis (76). Several lines of evidence sustain that PCSK9 promotes plaque formation in mice and human (29, 67, 77). Indeed, PCSK9 increases LDL uptake by macrophages scavenger receptors contributing to cell foam formation (50); it favors inflammation at the atherosclerotic vascular wall by inducing the expression of adhesion molecules, chemoattractants and inflammatory cytokines (78) and it induces ECs apoptosis reducing vessel stability (69). Furthermore, increased PCSK9 expression levels are associated to low shear stress (60, 61). Therefore, PCSK9 is an efficient target for the development therapies toward the prevention and treatment of atherosclerotic plaque formation.

Anti-PCSK9 therapy in mice reduced by half the plaque area in the aortic root, and the infiltration of pro-inflammatory macrophages in the atherosclerotic plaque was decreased (79). Serum levels of CXCL1, CXCL3, and CXCL10 (known chemoattractants for leukocytes), mainly produced by ECs and VSMCs, were reduced (79). Also, *Pcsk9* knockout mice show reduced expression of vascular cell adhesion molecule 1 (VCAM-1), a protein needed for immune cell adhesion to the vascular wall (57).

Anti-PCSK9 vaccination is an alternative to monoclonal antibody therapy. Vaccination involves the conjugation of a peptide (8–13 amino acids) that mimics the N-terminal domain of mature PCSK9 to a carrier protein that confers immunogenic properties to activate the immune system. Syntheses of host specific antibodies against PCSK9 generating long-term inhibition are obtained. Vaccination therapy aims to overcome monoclonal antibody therapy disadvantages including short *in vivo* half-lives, frequent dosage administration and high costs (80). In atherosclerosis mice models, inhibition of PCSK9 activity through vaccination decreased the expression of intercellular adhesion molecule 1 (ICAM-1) in the diseased aortic root and consequently there was a reduction in monocyte adhesion and migration to the endothelium that contributed to a reduction in atherosclerotic lesions (81). The anti-PCSK9 vaccine AT04A, which generates persistent humoral immune response against PCSK9 for 1 year in mice, reduced LDL content by more than 50% (81). It also reduced NLRP3 inflammasome expression in macrophages (81), a powerful inducer for PCSK9 expression and secretion in macrophages needed for the formation and progression of atherosclerotic plaques (51).

In humans, PCSK9 inhibitors therapy added to statin therapy is capable of increasing fibrous cap thickening in acute coronary syndrome patients, reducing plaque vulnerability (82). However, the LDL-cholesterol lowering capacities of both PCSK9 inhibitors and statin treatment cannot solely explain the increased fibrous cap thickness suggesting that an unknown pleiotropic effect such as an anti-inflammatory effect independent of lowering LDL-cholesterol may be involved (82). PCSK9 inhibitor treatment in an atherogenic mouse model increased the number of circulating endothelial progenitor cells and circulating angiogenic cells, markers of endothelial and vascular health associated with positive outcomes as reduced occurrence of cardiovascular events and death associated to cardiovascular causes (83).

## Role of PCSK9 in Inflammation in the Adaptive Immune System

The role of PCSK9 during atherosclerosis progression in the adaptive immune system has been studied. Dendritic cells (DCs) and T lymphocytes are localized in the atherosclerotic plaque, usually at sites prone to rupture (84). DCs mainly work as antigen presenting cells to T lymphocytes. They phagocyte antigens and present them to T lymphocytes in a process that involves MHC and TCR complexes (in DCs and T lymphocytes, respectively). In atheroma plaques, DCs present oxLDL fragments to T-lymphocytes that are then activated (85). The importance of T cell activation in atherosclerosis was demonstrated because *ApoE* knockout and immunodeficient (severe combined immunodeficiency mice without functional B and T lymphocytes) mice had less atherosclerotic lesions than *ApoE* knockout mice alone. Furthermore, CD4 T lymphocytes from *ApoE* knockout mice transferred to immunodeficient *ApoE* knockout mice induce the generation of atherosclerotic lesions (86). PCSK9 is induced by oxLDL in DCs and enhances the expression of proteins involved in T cell activation including CD80, CD83, CD86, and HLA-DR and the production of pro-inflammatory cytokines including TNFα, IL-1β, and IL-6. The expression of all these proteins was reduced when PCSK9 was silenced, and TGFβ and IL-10 expression levels were increased (87). T cells activated by oxLDL-stimulated DCs produced mainly IFNγ and IL-17, indicating a polarization toward an anti-inflammatory Th1/Th17 phenotype. These anti-inflammatory T regulatory cells inhibit foam cell formation and reverse the pro-inflammatory phenotype of macrophages reducing atherosclerosis progression (88). PCSK9 is a key molecule in Th17 response as atherosclerotic *Pcsk9/Ldlr/Apobec* (apolipoprotein B mRNA-editing catalytic polypeptide-1) triple knockout mice had significant lower Th17 production in comparison with atherosclerotic *Ldlr/Apobec* double knockout mice. This was associated with changes in the different cellular sources of Th17 (Th17 lymphocytes or γδTCR^+^ T cells). Indeed, mice lacking PCSK9 had a reduced number of Th17 lymphocytes as well as a reduced expression of RORγT, the transcription factor needed for Th17 lymphocyte differentiation (89).

A very recent work shows that PCSK9 downregulates the expression of MHC class I proteins in tumor cells by promoting its internalization and degradation in lysosomes (in a similar manner to that of PCSK9 with LDLR). Therefore, PCSK9 decreases the cytotoxic T lymphocyte response against the tumor (90). In an atherosclerotic context it seems plausible that modified LDLs could stimulate antigen presenting cells such as DCs or B cells to produce a variety of cytokines that would guide T lymphocytes differentiation toward a particular inflammatory subtype.

## PCSK9's Role in Familial Hypercholesterolemia

In 2003, after the discovery of PCSK9 gain-of-function mutations in FH patients the first monoclonal antibodies against PCSK9 were tested in preclinical and clinical studies (including ODYSSEY LONG TERM, ODYSSEY OUTCOMES, and FOURIER) demonstrating efficacy in reducing LDL cholesterol plasma levels in patients (91). In 2020 a recent sub analysis of the FOURIER and ODYSSEY OUTCOMES trials revealed that PCSK9 inhibition in patients with stable atherosclerosis and hyperlipidemia on statin therapy significantly reduces the risk of venous thromboembolism supporting a protective role for antiPCSK9 antibodies in human cardiovascular diseases (92). Monoclonal antibodies against PCSK9 are also capable of reversing the pro-inflammatory phenotype of atherogenic macrophages in patients with FH. PCSK9 inhibitors reduced CCR2, CX3CR1, and integrins CD11b and CD18 expression in circulating monocytes suggesting a lower infiltrating and chemoattractant capacity. PCSK9 antibody treatment reduced the production of TNFα by monocytes while the production of anti-inflammatory cytokine IL-10 was enhanced (93). In fact, circulating monocytes from FH patients were enriched with lipid droplets despite non-detectable LDLR expression but increased expression of CD36 and SRA [Figure 1; (93)]. Also, ABCA1 protein, a protein responsible for cholesterol efflux in macrophages, was inhibited upon PCSK9 expression (94). Taken together, these results suggest that circulating monocytes are pre-conditioned in FH patients due to PCSK9 activity, which enhances their infiltrating capacity, lipid accumulation and pro-inflammatory activity. Indeed, a prospective study with heterozygous FH patients under standard statin therapy revealed a positive correlation between circulating levels of PCSK9 and adverse cardiovascular events (95).

## PCSK9 and Platelet Thrombosis

Several risk factors associated with cardiovascular disease, including hyperlipidaemia, induce endothelial dysfunction and lead to arterial or venous thrombosis (96). In arteries with ongoing atherosclerosis progression, atherosclerotic plaque rupture is the main cause for thrombosis (97).

**Figure 2:**
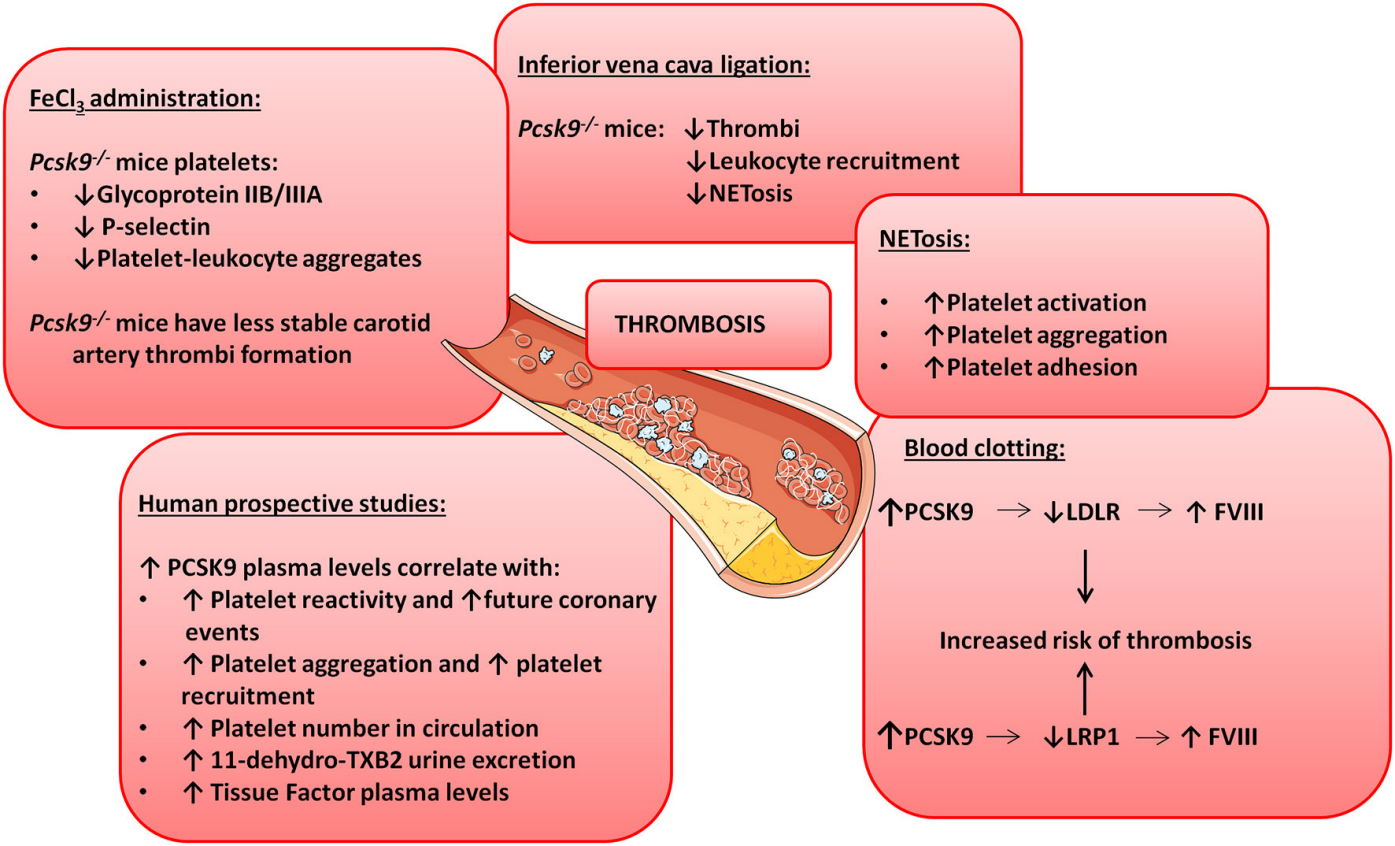
PCSK9 aggravates thrombosis. Schematic showing the involvement of PCSK9 in different thrombotic processes.

*Pcsk9* knockout mice show reduced carotid artery thrombosis induced by FeCl_3_ (a technique to rapidly and accurately induce thrombi formation) in different sized arteries and veins (98). Upon FeCl_3_ stimulation, 70% of *Pcsk9* knockout mice developed non-occlusive non-stable thrombi after 30 min while 57% of *Wt* mice showed total artery occlusion before 15 min after FeCl_3_ administration suggesting a role for PCSK9 in platelet reactivity (98). Furthermore, platelets from *Pcsk9* knockout mice show a significant reduction in glycoprotein IIB/IIIA expression levels, P-selectin expression levels and in circulating platelet-leukocyte aggregates in comparison with *Wt* mice indicating lower platelet activation in *Pcsk9* knockout mice (98). Similarly, *Pcsk9* knockout mice also show reduced thrombi formation after inferior vena cava ligation in comparison to *Wt* mice (99). Thrombi generated by inferior vena cava ligation in *Pcsk9* knockout mice have less leukocyte attachment as leukocyte recruitment is dependent on P-selectin and CXCL1, which are downregulated in *Pcsk9* knockout mice (100). However, it is unknown whether this inflammatory cell recruitment is downregulated because of PCSK9's role in lipid uptake or because PCSK9 has lipid-independent functions on platelet's steady-state (Figure 2 illustrates some of the mechanisms by which PCSK9 induces thrombosis).

NETosis is the process by which neutrophils release their nuclei content composed of DNA and antimicrobial proteins including neutrophil elastases and histones, creating networks of extracellular fibers that trap and facilitate the killing of pathogens (101). NETosis is linked to thrombosis because it causes platelet activation, aggregation and adhesion (102) and promotes the initiation of the coagulation cascade (100). In *Pcsk9* knockout mice NETosis is significantly reduced, despite that the total number of blood neutrophils and leukocytes are increased suggesting that PCSK9 can induce thrombosis by stimulating NETosis [Figure 2; (99)].

The PCSK9-REACT study is an observational, prospective study where patients with recent acute coronary syndromes underwent coronary intervention and received P2Y_12_ inhibitors (103). P2Y_12_ is a chemoreceptor for adenosine diphosphate (ADP) involved in platelet aggregation (104) and a target for thromboembolism treatments using antagonists as ticagrelor or prasugrel (105). The study revealed a strong correlation between PCSK9 blood levels and platelet reactivity (103). It also showed that elevated PCSK9 plasma levels are associated with future coronary events as 22% of patients with the highest PCSK9 plasma levels suffered coronary events while only 2% of the patients in the lower tertile experienced coronary events. In line with these results, human recombinant PCSK9 added to healthy human plasma was capable of significantly increasing platelet aggregation and reducing aggregation lag time when platelets were stimulated with epinephrine (98). The platelet enhancing capacity is because addition of PCSK9 increased the total number of platelets that express the activation marker glycoprotein IIB/IIIA by 36% [Figure 2; (98)].

A relationship between PCSK9 plasma levels and total number of circulating platelets has also been shown in patients with stable coronary artery disease (106). Similarly, atrial fibrillation patients show a strong correlation between PCSK9 plasma levels and platelet reactivity as elevated PCSK9 levels positively correlate with elevated risk for this cardiovascular event (107). These patients also have higher rate of platelet aggregation and recruitment coincidentally with higher expression levels of thromboxane B_2_ (TxB_2_, a platelet activation marker), higher release of P-selectin and enhanced ROS formation (108). Correlation between elevated PCSK9 plasma levels and elevated urine excretion of TxB_2_ was also found (108). Taken together, these results show that not only platelet number but also platelet reactivity is enhanced when PCSK9 plasma levels are elevated (Figure 2).

Cholesterol incorporation into platelet membranes induces platelet reactivity while cholesterol depletion from membranes is associated with platelet stability (109). Thus, PCSK9 inhibition would decrease plasma LDL levels reducing platelet reactivity. As a matter of fact, statins treatment in hypercholesterolemic patients, is able to reduce platelet membrane cholesterol (110). It remains a matter of discussion whether PCSK9 exerts a direct effect on platelets or the effects depend on the dyslipidaemia generated by PCSK9 binding to LDLR. Dyslipidaemia induces the generation of oxLDL and agLDL, which in turn, facilitate platelet activation by binding scavenger receptors on platelet's surface including LOX-1 and CD36 (111–113). Once activated, platelets are capable of oxidizing LDLs, generating a positive feedback of platelet activation (114). PCSK9 inhibition also downregulates lipoprotein (a) [Lp(a)] serum levels in patients with inherited dyslipidemias (115). Since Lp(a) enhances platelet activation and thrombosis (116–119), PCSK9 may prevent thrombosis by lowering Lp(a) levels. A direct effect of PCSK9 on platelets independent of its effects on dyslipidaemia has been recently shown as PCSK9 inhibitors can enhance oxidative stress (as a result of the activation of the Nox2 and cPLA_2_ signaling cascades) and block platelet activation in *Wt* human platelets (108).

PCSK9 inhibitors are being tested to modulate platelet activation in humans. Patients with primary hypercholesterolemia with previous statin treatment were treated for 12 months with alirocumab or evolocumab and after only 2 months treatment a significant decrease in the platelet activation marker CD62P was found (120). Soluble CD40, soluble P-selectin and platelet factor 4 plasma levels were also reduced after 12 months of statin and PCSK9 inhibitor treatment. The study also shows that hypercholesterolemic patients with additional acetylsalicylic acid administration to statins and PCSK9 inhibitors have decreased platelet aggregation (120). A trend in the reduction of platelet aggregation in patients without acetylsalicylic acid administration was observed but there were no significant differences because of the low number of patients that followed this treatment (120).

PCSK9 is also involved in blood clotting. Clotting formation is a complex chemical process were circulating blood clotting factors will sequentially induce protein cleavages to generate thrombin and fibrin (121). A correlation between elevated blood clotting Factor VIII (FVIII) plasma levels and arterial thrombosis has been shown in both animal and human studies (122–124). FVIII synthesis and clearance (and therefore FVIII plasma levels) are regulated by the liver. Indeed, LDLR and LRP1 expressed in hepatocytes promote FVIII endocytosis and degradation (125–128). Although not demonstrated yet, a connection between PCSK9 and FVIII seems plausible. Indeed, downregulation of LDLR expression in hepatocytes cell surface regulated by PCSK9 induces an increase in FVIII plasma levels and therefore an increased risk of thrombosis and cardiovascular events (127). PCSK9 can also reduce LRP1 cell surface expression further increasing FVIII plasma levels (53, 129). Finally, in patients that produce anti-phospholipidic antibodies, polymorphisms in PCSK9 and LDLR genes are associated with thrombosis progression supporting a role in clotting formation for the PCSK9-LDLR axis [Figure 2; (130)].

## Concluding Remarks

Since PCSK9 was first described as the inducer of some FH pathologies, a lot of interest has been placed in the achievement of an effective inhibitory treatment. PCSK9 inhibitors administered to patients revealed a key role of PCSK9 in atherosclerotic disease as its inhibition reduced plasma LDL-cholesterol levels with improved clinical cardiovascular outcomes demonstrating a multifactorial and pathophysiological role for PCSK9 in atherosclerosis progression. Interestingly, PCSK9 functions are far from only regulating LDL-cholesterol plasma levels by reducing hepatic LDLR expression. Indeed, recent findings demonstrate that PCSK9 is also actively modulating inflammation, plaque formation and thrombosis. Hence, the benefits observed from PCSK9 inhibitory therapies may not only be induced by its plasma lipid-lowering capacities but also by reducing the impact of several other mechanisms in which PCSK9 is involved that are actively promoting atherosclerosis. Unfortunately, the information on PCSK9 interactome is still limited and further investigations on the role of PCSK9's activity on different signaling pathways are still needed to generate a clear vision of PCSK9 full potential during atherosclerosis progression. Despite PCSK9 has been studied mostly in cardiovascular diseases, it also participates in general mechanisms shared by many other diseases, and hence it is conceivable that PCSK9 is involved in the initiation and progression of other pathologies with powerful inflammatory or thrombotic components.

## Author Contributions

AL: research performance, draft manuscript preparation, writing and review. MB-P: conceptualization, funding acquisition, draft manuscript preparation, writing, review, and editing. LB: conceptualization and funding acquisition. All authors contributed to the article and approved the submitted version.

## Conflict of Interest

The authors declare that the research was conducted in the absence of any commercial or financial relationships that could be construed as a potential conflict of interest.
